# Supplementary material for: A Qualitative Review of Patient Feedback for the OPAT (Outpatient Antimicrobial Therapy) Service in Bristol
Source: Antibiotics (Basel). 2024 May 3;13(5):420. doi: 10.3390/antibiotics13050420 (PMC11117369; doi:10.3390/antibiotics13050420)
Supplement: Supplementary file 1 [file antibiotics-13-00420-s001.zip › antibiotics-2782339-supplementary S1.docx]

**Outpatient Parenteral Antibiotic Therapy (OPAT)**

**Patient Feedback Survey**

You have recently received care via the OPAT service. The purpose of this survey is to capture your experience throughout your treatment. We welcome any constructive feedback. Please answer the questions below.

Name *(optional)*: Date:

| 1. | **Based on the care you received, how would you rate the service?** (Please circle as appropriate) | | | | | | | | | | | | | | | |
| --- | --- | --- | --- | --- | --- | --- | --- | --- | --- | --- | --- | --- | --- | --- | --- | --- |
|  | Poor service | | | | | | | | Excellent service | | | | | | | |
|  | 0 | 1 | | 2 | 3 | | 4 | 5 | | 6 | 7 | | 8 | 9 | | 10 |
|  | **Please explain the reason for your rating** | | | | | | | | | | | | | | | |
| 2. | **Were there any benefits of the service to yourself, family, relatives or friends? If so, please specify** | | | | | | | | | | | | | | | |
| 3. | **Was the treatment plan explained to you by either the pharmacy or nursing team, prior to discharge?** | | | | | | | | | | | | | | | |
| 4. | **Do you feel you had appropriate support by the pharmacy and/or nursing team throughout your treatment?** | | | | | | | | | | | | | | | |
| 5. | **Please state any potential improvements that could be made to the service** | | | | | | | | | | | | | | | |
| 6. | **How likely are you to recommend the service to friends and family?** (please circle appropriate statement) | | | | | | | | | | | | | | | |
|  | Don’t Know | | Extremely Unlikely | | | Unlikely | | | Neither likely or unlikely | | | Likely | | | Extremely Likely | |
| 7. | **Any other comments?** | | | | | | | | | | | | | | | |
